# Fetal and neonatal effects of N-acetylcysteine for maternal chorioamnionitis: a systematic review

**DOI:** 10.3389/fped.2026.1819220

**Published:** 2026-09-02

**Authors:** Fawzia Mohamed Elgharbawy, Mohammad A. A. Bayoumi, Kenni W. Ajele, Bashir A. Lwaleed

**Affiliations:** 1 Neonatology Department, Faculty of Health Sciences, University of Southampton, Southampton, United Kingdom; 2 Neonatal Intensive Care Unite (NICU), Women’s Wellness and Research Center (WWRC), Hamad Medical Corporation (HMC), Doha, Qatar; 3 Faculty of Humanities, North-West University, Potchefstroom, South Africa

**Keywords:** cerebrovascular coupling, chorioamnionitis, fetal inflammatory response, intra-amniotic infection, N-acetylcysteine, neuroprotection, pharmacokinetics, preterm birth

## Abstract

**Background:**

Maternal chorioamnionitis (mCA) and intra-amniotic infection/inflammation (Triple I) are major causes of fetal inflammatory injury and are associated with preterm birth, white matter injury, cerebral palsy, bronchopulmonary dysplasia (BPD), and necrotising enterocolitis (NEC). No established therapy directly targets the inflammatory and oxidative pathways underlying fetal neuroinjury. N-acetylcysteine (NAC), a glutathione precursor with antioxidant and anti-inflammatory properties, has emerged as a potential adjunctive neuroprotective therapy, although human evidence remains limited.

**Objective:**

To evaluate fetal and neonatal outcomes associated with antenatal or intrapartum NAC administration in pregnancies complicated by mCA or Triple I.

**Methods:**

A systematic review was conducted using MEDLINE, Embase, Cochrane CENTRAL, and ClinicalTrials.gov from inception to December 23, 2025. Eligible studies included randomised controlled trials, prospective cohorts, non-randomised studies, and translational animal models evaluating NAC exposure in mCA or Triple I. Clinical outcomes, biomarkers, pharmacokinetics, and mechanistic pathways were synthesised narratively. Risk of bias was assessed using RoB 2, ROBINS-I, and SYRCLE tools.

**Results:**

Ten studies met the inclusion criteria, including five human and six translational animal studies. Human evidence involved approximately 143 mother–infant dyads. NAC exposure was associated with preserved cerebrovascular coupling, improved delivery-room adaptation, reduced severe neonatal morbidity, and lower BPD rates without major safety concerns. Pharmacokinetic studies demonstrated rapid placental transfer and gestation-dependent neonatal clearance. Animal studies consistently showed reductions in inflammatory cytokines, oxidative stress, apoptosis, and microstructural brain injury.

**Conclusion:**

NAC demonstrates biologically plausible neuroprotective potential in mCA and Triple I; however, the evidence remains preliminary due to small sample sizes and heterogeneous protocols. However, some studies showed no or negative effects. Large multicenter randomised trials with standardised dosing and long-term neurodevelopmental follow-up are needed before routine clinical implementation can be recommended.

## Introduction

Maternal chorioamnionitis (mCA) and intra-amniotic infection/inflammation (Triple I) are major causes of fetal inflammatory response syndrome and adverse neonatal outcomes, with microbial invasion and intra-amniotic inflammation representing central drivers of preterm inflammatory injury (1). These conditions are strongly associated with preterm birth and severe neonatal complications, including white matter injury (WMI), intraventricular haemorrhage (IVH), cerebral palsy (CP), bronchopulmonary dysplasia (BPD), and necrotising enterocolitis (NEC) (1–3). Chorioamnionitis complicates approximately 2%–5% of term births and up to 40% of extremely preterm deliveries (1, 2). Despite antibiotics and expedited delivery, inflammatory and oxidative pathways may persist, contributing to ongoing fetal and neonatal injury (3).

The pathophysiology of mCA involves activation of placental and fetal innate immune pathways following exposure to pathogen-associated molecular patterns such as lipopolysaccharide (LPS). This stimulates nuclear factor kappa B (NF-κB) signalling and release of pro-inflammatory cytokines, including IL-1β, IL-6, and TNF-α, leading to oxidative stress, endothelial dysfunction, impaired cerebrovascular autoregulation, and injury to vulnerable pre-oligodendrocytes involved in white matter maturation (2, 4, 5).

Current neuroprotective strategies remain limited. Magnesium sulfate reduces the risk of CP in imminent preterm birth but has inconsistent efficacy in established chorioamnionitis and does not directly target oxidative stress pathways (4). Therapeutic hypothermia is generally unsuitable for extremely preterm infants, while agents such as erythropoietin and melatonin have limited obstetric safety data (5, 6). Consequently, there remains a need for adjunctive therapies targeting inflammation-mediated fetal injury.

N-acetylcysteine (NAC), a glutathione precursor and free-radical scavenger, has emerged as a biologically plausible neuroprotective candidate because of its antioxidant, anti-inflammatory, and potential epigenetic effects (6, 7). Experimental studies suggest that NAC may preserve cerebrovascular autoregulation, reduce apoptosis, stabilise white matter integrity, and attenuate cytokine-mediated injury (3, 7, 8). Pharmacokinetic studies further demonstrate rapid placental transfer, supporting effective fetal exposure during active intrauterine inflammation (7).

Although NAC has an established safety profile in obstetric medicine, evidence regarding its neuroprotective efficacy in pregnancies complicated by mCA or Triple I remains fragmented and largely limited to small clinical and translational studies. Therefore, this systematic review aimed to comprehensively evaluate the fetal and neonatal effects of antenatal or intrapartum NAC administration in maternal chorioamnionitis and Triple I, with emphasis on clinical outcomes, pharmacokinetics, inflammatory biomarkers, mechanistic pathways, and safety profiles.

### Pathophysiologic rationale for Use of NAC in chorioamnionitis

Pathogen-associated molecular patterns like lipopolysaccharide (LPS) attach to Toll-like receptors on amniotic, chorionic, and placental macrophages during maternal chorioamnionitis. The activation of NF-κB results in increased transcription of IL-1β, IL-6, TNF-α, and chemokines into the fetal circulation. Fetal glutathione depletion via mitochondrial and NADPH oxidase–derived reactive oxygen species increases oxidative stress, which can change redox-sensitive gene transcription. Pre-oligodendrocyte damage, blood–brain barrier alterations, and cerebrovascular autoregulation can result from these inflammatory and oxidative mechanisms. NAC directly donates cysteine, increasing glutathione formation, buffering reactive oxygen species, and affecting NF-κB activation. Sulfane sulfur species from NAC may change microglial activation thresholds in other ways. Cord: maternal ratios >1 indicate rapid placental transfer, which allows maternal dosing during the evolving inflammatory time course to swiftly expose fetal/maternal exposure and improve antioxidant capacity in the postpartum period (7).

### Clinical burden and unmet need

The burden of chorioamnionitis is worsened in the setting of prematurity, with approximately 50% of very preterm infants exposed to chorioamnionitis going on to develop at least some degree of neurologic or pulmonary morbidity (8). Antibiotics and delivery are the standard treatments, which focus on resolving the infectious burden, but not the downstream and prolonged neuroinflammatory and oxidative sequelae (3). Since the evidence for magnesium sulfate is modest and context-dependent, and other potential neuroprotectants like erythropoietin or melatonin have limited obstetric safety data, NAC may be a pragmatic candidate to fill this gap (3, 6–8).

### Definitions and diagnostic criteria

Chorioamnionitis definitions vary by diagnosis. Maternal fever, in addition to at least 2 of maternal leukocytosis, uterine discomfort, fetal tachycardia, and foul/purulent amniotic fluid, is usually required. The Triple I (microbiologic infection, inflammation, and intra-amniotic cytokines) criteria add microbiologic confirmation (via amniotic fluid Gram stain or culture) to distinguish isolated maternal fever from true infection/inflammation. Histologic chorioamnionitis, identified on placental pathology after delivery, involves neutrophil infiltration in the membranes and chorionic plate but is not a real-time diagnostic. Different definitions affect research populations, pathogens seen, and baseline risk, emphasising the need for a more consistent inclusion criterion in future NAC trials (9).

### Current standard of care

Empiric broad-spectrum antibiotics, induction/augmentation, antipyretics, and fetal monitoring treat suspected chorioamnionitis. Despite minimal studies on intrauterine infection, magnesium sulfate may be given for neuroprotection before early preterm birth. Fetal lung maturation would continue with antenatal corticosteroids. These precautions do not eliminate residual BPD, NEC, and neurologic damage, leaving a place for adjuncts such as NAC that target oxidative and inflammatory pathways (10).

### Aim and eligibility criteria

- **Aim:** This comprehensive study assesses the safety and efficacy of NAC for neuroprotection in prenatal, intrapartum, or early neonatal pregnancies with maternal chorioamnionitis or intra-amniotic infection/inflammation.
- **Study types:** The study types include RCTs, NRCTs, prospective cohorts, and translational animal studies published in English. Studies fully covered by past systematic reviews and abstract-only reports were removed.
- **Interventions:** Oral or intravenous NAC for neuroprotection in pregnant individuals with chorioamnionitis/Triple I, with or without early neonatal dose. Studies of pregnant NAC patients for unrelated disorders were excluded.

### Comparators: placebo, standard care, or no NAC

Outcomes: Clinical outcomes (fetal/neonatal mortality, severe composite morbidity, BPD, NEC, cerebrovascular regulation, neuroimaging, neurodevelopment), pharmacokinetics, and mechanistic biomarkers (cytokines, oxidative stress, NF-κB, apoptosis).

## Methods

### Study design and reporting framework

**Figure 1:**
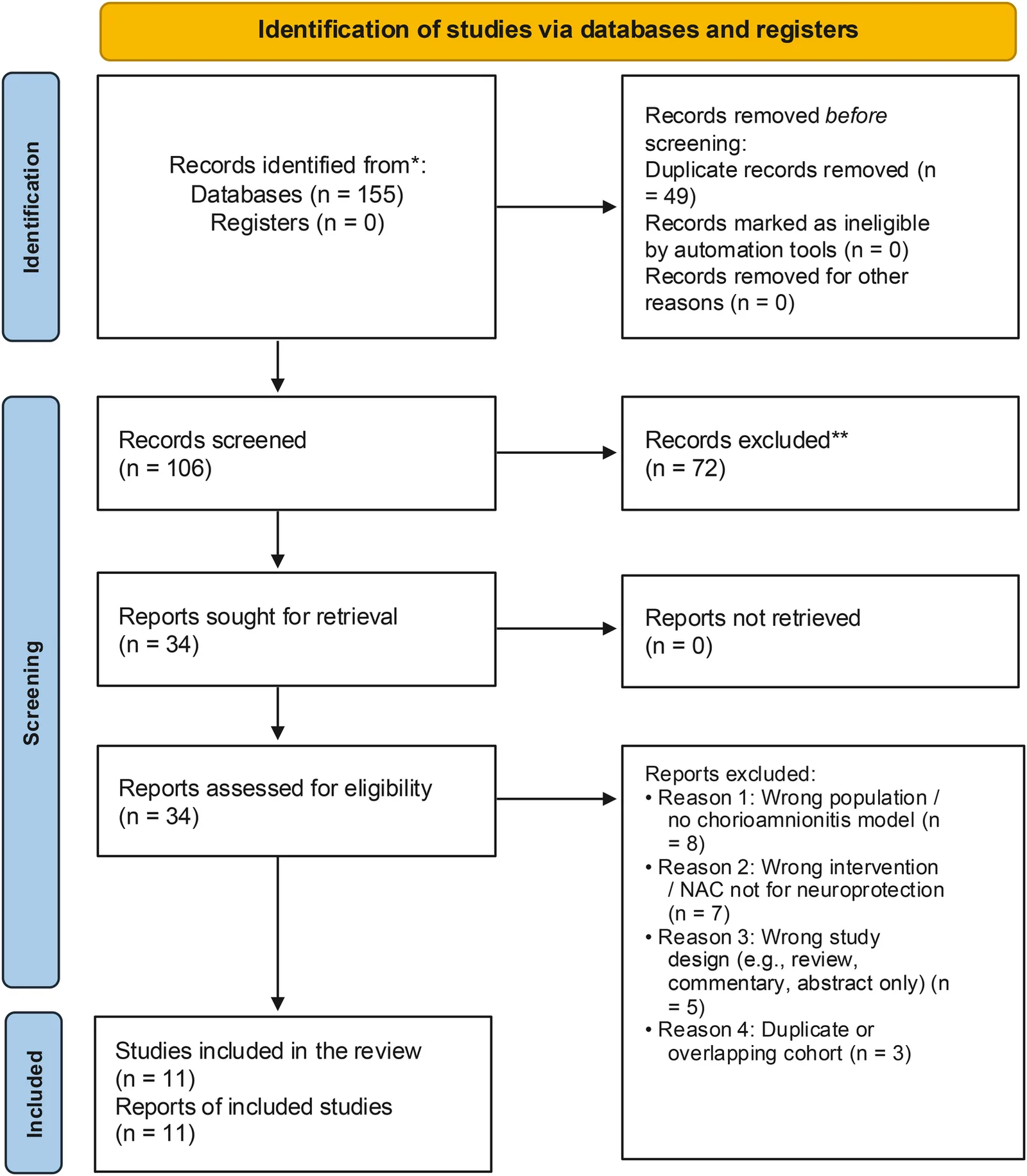
PRISMA flow diagram of study selection process.

This systematic review was conducted in accordance with the Preferred Reporting Items for Systematic Reviews and Meta-Analyses (PRISMA) guidelines (Figure 1) to evaluate the fetal and neonatal effects of N-acetylcysteine (NAC) in pregnancies complicated by maternal chorioamnionitis (mCA) or intra-amniotic infection/inflammation (Triple I). Human clinical studies and translational animal models assessing neuroprotective outcomes, pharmacokinetics, inflammatory biomarkers, and mechanistic pathways were included.

Figure 1 shows that this systematic review follows PRISMA study selection guidelines. The literature search found all human and translational animal studies on N-acetylcysteine (NAC)'s neuroprotective effects in maternal chorioamnionitis or intra-amniotic infection/inflammation (Triple I From inception to December 23, 2025, PubMed, Embase, CENTRAL, and ClinicalTrials.gov were searched without date, language, publication status, or study type restrictions. Search queries used NAC, chorioamnionitis, and neuroprotection-controlled keywords. RCTs, non-RCTs, prospective cohorts, and mechanistic animal studies were relevant. Deduplication was performed using electronic search references in Covidence systematic review software (Veritas Health Innovation, Melbourne, Australia). The title, abstract, and full-text screening were done independently by two investigators, with inconsistencies addressed by consensus. A second author examined predetermined research characteristics, interventions, outcomes, and other findings. The methodological quality and risk of bias of the publications were assessed using Cochrane RoB 2 for RCTs and SYRCLE's animal study risk-of-bias recommendations. Due to projected clinical and methodological variability amongst studies, a narrative synthesis was performed, and a summary table was created for effectiveness, safety, and mechanism comparisons.

The qualitative synthesis included 11 studies. Five papers included humans, but only three were original clinical research cohorts (7, 10, 11). A safety follow-up of the Jenkins et al. randomised controlled trial (11) and a neonatal pharmacokinetic subset analysis from the Wiest et al. study (7) were secondary studies and did not represent independent study populations. Six further preclinical animal models represented different types of experiments (4, 12–16). The clinical evidence base is fundamentally derived from three principal human studies, supplemented by mechanistic and translational data from six animal studies.

### Search strategy

A comprehensive and systematic literature search was conducted in MEDLINE (PubMed), Embase, Cochrane CENTRAL, and ClinicalTrials.gov from database inception to December 23, 2025, to identify studies evaluating the fetal and neonatal effects of N-acetylcysteine (NAC) in maternal chorioamnionitis (mCA) or intra-amniotic infection/inflammation (Triple I). The search strategy combined controlled vocabulary terms (e.g., MeSH terms) with free-text keywords related to NAC, chorioamnionitis, neuroprotection, fetal inflammation, and prematurity. Boolean operators (“AND,” “OR”) and truncation techniques were applied to optimise search sensitivity and specificity. The search included terms related to NAC (“N-acetylcysteine,” “Acetylcysteine,” “NAC”), maternal and intra-amniotic inflammation (“chorioamnionitis,” “Triple I,” “intra-amniotic infection,” “funisitis,” “intrauterine inflammation”), neuroprotection (“neuroprotection,” “brain protection,” “neurodevelopment,” “cerebral blood flow”), and prematurity (“preterm infant,” “premature neonate,” “infant, premature”). Controlled vocabulary terms included MeSH headings such as “Acetylcysteine,” “Chorioamnionitis,” “Fetus,” and “Infant, Premature.” A sample PubMed search strategy was: (“acetylcysteine” OR “N-acetylcysteine” OR “NAC”) AND (“chorioamnionitis” OR “Triple I” OR “intra-amniotic infection”) AND (“fetus” OR “neonate” OR “preterm infant”) AND (“neuroprotection” OR “brain” OR “neurodevelopment”). Searches were adapted appropriately for each database.

### Study selection and eligibility criteria

All identified records were imported into Zotero for reference management and subsequently uploaded into Covidence systematic review software (Veritas Health Innovation, Melbourne, Australia) for deduplication and screening. Two reviewers independently screened titles and abstracts, followed by full-text assessment of potentially eligible studies. Disagreements were resolved through discussion and consensus. Reference lists of included studies were also manually screened to identify additional eligible records.

Studies were selected according to predefined eligibility criteria based on population, intervention, comparator, outcomes, and study design. Eligible human studies included pregnant individuals with clinical or microbiologically confirmed maternal chorioamnionitis (mCA) or intra-amniotic infection/inflammation (Triple I). Translational animal studies employing maternal inflammatory models relevant to fetal neuroinflammation were also included to support the mechanistic interpretation of NAC-mediated neuroprotection.

Eligible interventions included antenatal, intrapartum, or immediate neonatal administration of N-acetylcysteine (NAC) for fetal or neonatal neuroprotection. Comparators included placebo, standard care, or no NAC exposure. Studies were required to report neuroprotection-related outcomes, including cerebrovascular regulation, neuroimaging findings, neonatal morbidity, mortality, neurodevelopmental outcomes, pharmacokinetic parameters, inflammatory biomarkers, oxidative stress markers, or mechanistic pathways. Animal studies were included when they assessed biologically relevant neuroprotective outcomes, including cytokine expression, oxidative stress, apoptosis, MRI findings, or sensorimotor function.

Randomised controlled trials (RCTs), non-randomised controlled studies, prospective cohort studies, and translational animal studies published in English were included to capture the breadth of emerging evidence in this evolving field. Reviews, commentaries, abstract-only reports, duplicate datasets, and studies evaluating NAC for unrelated maternal or fetal indications were excluded because they did not provide sufficient primary outcome data relevant to the review's objectives.

### Data extraction and management

Data extraction was performed independently using a standardised data extraction form developed for this review. Extracted information included study design, study setting, sample size, gestational age, diagnostic criteria for maternal chorioamnionitis or Triple I, NAC dosing regimen, route and timing of administration, comparator characteristics, co-interventions, follow-up duration, and primary findings. Clinical outcomes extracted included neonatal morbidity, neurodevelopmental outcomes, cerebrovascular regulation, respiratory and gastrointestinal complications, and mortality. Pharmacokinetic studies provided data on neonatal clearance, half-life, volume of distribution, and cord-to-maternal concentration ratios. For translational animal studies, details regarding inflammatory models, timing of NAC administration relative to inflammatory exposure, and mechanistic biomarkers such as cytokine expression, oxidative stress markers, apoptosis, and MRI findings were recorded. Data extraction was designed to capture both clinical efficacy and mechanistic evidence supporting NAC-mediated neuroprotection in inflammation-associated fetal injury. Authors were not contacted for missing information, which was acknowledged as a limitation of the review.

### Risk of bias and certainty assessment

Risk of bias was assessed using study design–specific tools. Randomised controlled trials were evaluated using the Cochrane Risk of Bias 2 (RoB 2) tool, non-randomised human studies were assessed using ROBINS-I, and translational animal studies were assessed using SYRCLE risk-of-bias domains. The judgments considered randomisation, allocation concealment, deviations from intended interventions, missing outcome data, outcome measurement, selective reporting, and other potential sources of bias, as appropriate for each study design.

**Figure 2:**
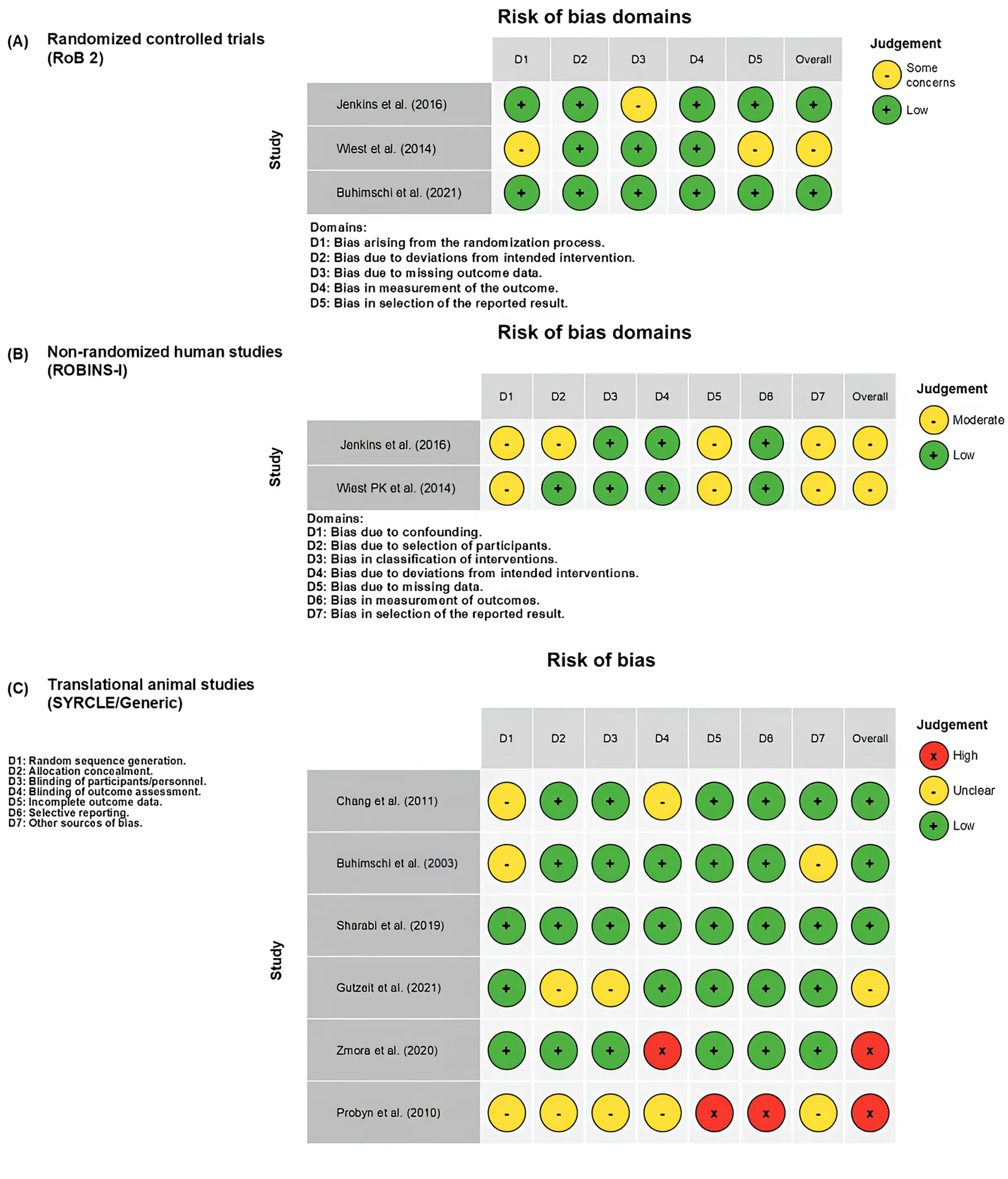
Risk-of-Bias assessment of included studies. **(A)** Randomised controlled trials assessed using the Cochrane Risk of Bias 2 (RoB 2) tool; **(B)** non-randomised human studies assessed using ROBINS-I; and **(C)** translational animal studies assessed using SYRCLE risk-of-bias domains. Risk-of-bias visualisations were generated using the *robvis* R package and Shiny web application (McGuinness & Higgins, 2020). RoB 2, Risk of Bias 2; ROBINS-I, Risk of Bias in Non-Randomized Studies of Interventions; SYRCLE, Systematic Review Centre for Laboratory Animal Experimentation.

Risk-of-bias visualisations were generated using the robvis R package and Shiny application. The results are presented separately for randomised controlled trials, non-randomised human studies, and translational animal studies in Figures 2A–C. The overall certainty and applicability of the evidence were considered narratively using GRADE domains, including risk of bias, inconsistency, indirectness, imprecision, and publication bias. Formal certainty ratings were not assigned because the evidence was synthesised narratively without quantitative pooling (Table 1 and Figure 2).

**Table 1:** Summary of risk of bias assessments.

**Study**	**Design**	**Tool**	**Risk of Bias**	**Key Strengths**	**Key Limitations**
Jenkins et al. (11)	Double-blind RCT	RoB 2	Low risk	Double-blind, complete follow-up, blinded outcome assessment	Single-centre, modest sample size
Wiest et al. (7)	PK RCT	RoB 2	Some concerns	Objective PK outcomes, blinded lab assessment	Unclear allocation concealment
Buhimschi et al. (10)	Quadruple-blind RCT	RoB 2	Low risk	Quadruple-blind, rigorous Triple I definition, large effect size	Single center
Chang et al. (12)	Mouse LPS model	SYRCLE	Unclear	Multiple outcomes (cytokines, myelin)	Incomplete reporting of blinding
Buhimschi et al. (4)	Mouse model	SYRCLE	Unclear	Foundational study, GSH measurements	Incomplete reporting of methods
Sharabi et al. (13)	Rat MRI model	SYRCLE	Low risk	MRI outcomes, blinded assessors	Only male offspring were studied
Gutzeit et al. (14)	Rat hypoxia model	SYRCLE	Low risk to some concerns	Multiple outcomes, blinded analysis	Allocation concealment unclear
Zmora et al. (15)	Rat NEC model	SYRCLE	Low risk to some concerns	Large sample, blinded histology	Allocation concealment unclear
Jenkins et al. (follow-up) (11)	Cohort follow-up	ROBINS-I	Moderate risk	Non-randomised follow-up; attrition and selection bias	33% loss to follow-up
Wiest et al. (PK subset) (7)	Sub-analysis	ROBINS-I	Moderate risk	Small non-random subset; confounding and selective reporting	Restricted sample size, GA/maturity confounding variables, no clinical outcome data.
Probyn, et al. (16)	Controlled *in vivo* animal experiment in chronically catheterised preterm fetal sheep.	SYRCLE	High risk of bias.	− Four-group controlled design, including LPS-only and NAC-only controls − Objective serial measurement of blood gases, blood pressure, heart rate, hematology, and TNF-alpha. − Standardized catheterization and treatment schedule with repeated-measures analysis. − Baseline data, animal ethics approval, and funding were reported.	− Very small and unequal groups, especially NAC *n* = 3; no sample-size calculation reported. − No reported randomisation, allocation concealment, random housing, or blinding. − Different NAC doses were pooled, limiting dose-response interpretation. − Data-dependent time windows, multiple *post-hoc* tests, some missing heart-rate data, and limited human applicability because NAC was administered directly to fetal sheep.

### Synthesis and analysis

Given the variability in NAC dosing regimens and the timing (prophylactic vs. therapeutic), populations (preterm vs. near-term), and outcomes, meta-analytic pooling was not attempted. A structured narrative synthesis was performed, supported by a comparative summary table. Clinical outcomes were prioritised for presentation, followed by PK and mechanistic studies. Safety signals were noted separately. Where possible, absolute and relative risk differences reported by study authors were transcribed to enable indirect comparison across studies. No missing data imputation was performed.

### Inclusion and exclusion criteria

- **Population:** We included pregnant individuals older than 18 years of age with clinical or microbiologically confirmed chorioamnionitis/Triple I (including those with fetal status perturbation) at GA between 23 and 32 weeks. Mono- or polymicrobial maternal inflammation model in animals.
- **Study types** Only systematic reviews, cohort studies, RCTs, and NRCTs published in English will be included. Those studies published only as abstracts will be excluded. Trials that were already included in previous systematic reviews will be excluded.
- **Intervention:** Antenatal, intrapartum, or immediate neonatal period administration of NAC with the intent of neuroprotection.
- **Comparator:** Placebo or no NAC. Concomitant antibiotics and routine obstetric/neonatal care were permitted.
- **Outcomes:** All neuroprotection-relevant outcomes (including cerebrovascular regulation, neuroimaging, and developmental outcomes), as well as respiratory and gastrointestinal morbidities, mortality, PK parameters, and mechanistic biomarkers.

### Certainty in evidence and applicability

The risk of bias, inconsistency, indirectness, imprecision, and publication bias was qualitatively assessed but not rated. GRADE domains contextualised certainty, but no grades were given. We did not plan meta-analyses or pool results; thus, GRADE scores were not given. Dosing regimen feasibility, diagnostic criteria alignment with Triple I definition, and safety monitoring were utilised to assess applicability to current practice.

### Outcomes and definitions

The primary outcomes were neuroprotection-related fetal and neonatal outcomes, including cerebrovascular coupling, severe composite neonatal morbidity, neuroimaging abnormalities, and neurodevelopmental impairment. Composite severe neonatal morbidity included death, severe intraventricular haemorrhage, periventricular leukomalacia, necrotising enterocolitis stage II or higher, and severe bronchopulmonary dysplasia.

Secondary outcomes included Apgar scores, delivery-room resuscitation requirements, ventilator duration, neonatal intensive care unit stay, respiratory and gastrointestinal morbidity, and mortality. Additional mechanistic outcomes included inflammatory cytokines (e.g., IL-1β, IL-6, TNF-α), oxidative stress markers, apoptosis-related pathways, and epigenetic markers such as HDAC2 expression.

Pharmacokinetic outcomes included maternal and neonatal clearance, half-life, volume of distribution, and cord-to-maternal concentration ratios. These outcomes were selected to comprehensively evaluate the clinical, biological, and mechanistic effects of NAC in inflammation-mediated fetal and neonatal injury.

### Data synthesis and statistical analysis

A structured narrative synthesis was conducted to integrate findings from the included human and translational animal studies. Extracted data were organised according to study design, population characteristics, NAC dosing regimen, timing of administration, comparator groups, and reported outcomes. Clinical findings were synthesised descriptively across studies, with particular focus on neuroprotective outcomes, severe neonatal morbidity, respiratory and gastrointestinal complications, pharmacokinetic parameters, inflammatory biomarkers, and mechanistic pathways.

Human studies were summarised comparatively according to maternal and neonatal characteristics, intervention protocols, and outcome measures. Pharmacokinetic findings, including placental transfer, neonatal clearance, half-life, and volume of distribution, were synthesised separately because of their clinical relevance for dosing considerations. Translational animal studies were reviewed qualitatively to examine the mechanistic effects of NAC on inflammatory cytokines, oxidative stress, apoptosis, white matter injury, and neuroimaging abnormalities.

Because of substantial heterogeneity in study designs, sample characteristics, NAC administration protocols, outcome definitions, and follow-up duration, findings were synthesised descriptively rather than quantitatively pooled. Reported relative risks, confidence intervals, and other descriptive outcome measures were extracted and summarised where available to facilitate comparison across studies. No imputation of missing data was performed.

## Results

### PRISMA flow diagram

The study selection process is presented in Figure 1. A total of 155 records were identified through database searching. After removal of 49 duplicate records, 106 studies underwent title and abstract screening, of which 72 were excluded. Thirty-four full-text reports were assessed for eligibility, and 23 were excluded because of the wrong population or absence of a chorioamnionitis model (*n* = 8), inappropriate intervention or NAC not administered for neuroprotection (*n* = 7), unsuitable study design, such as reviews or abstract-only publications (*n* = 5), and duplicate or overlapping cohorts (*n* = 3). Ultimately, 11 studies met the inclusion criteria and were included in the final qualitative synthesis (Figure 1). No negative human or preclinical studies were excluded due to negative findings.

### Characteristics of included studies

Characteristics of the included studies are summarised in Table 2. The review included 10 studies comprising five human studies and six translational animal studies. Human studies consisted of three randomised controlled trials, one prospective cohort extension, and one neonatal pharmacokinetic sub-study, all conducted in the United States. Sample sizes ranged from 11 to 67 mother–infant dyads, and NAC was administered antenatally, intrapartum, postnatally, or in combined maternal-neonatal protocols. Primary clinical outcomes included cerebrovascular coupling, severe neonatal morbidity, bronchopulmonary dysplasia (BPD), pharmacokinetic parameters, and long-term neurodevelopmental outcomes (Table 2).

**Table 2:** Characteristics of All included studies.

Study	Design	Setting/Model	Population/Species	Intervention (NAC Regimen)	Comparator	Primary Outcomes	Key Findings
Jenkins et al. (11)	Double-blind RCT	Single centre (USA)	22 mothers, 24 infants with clinical chorioamnionitis	Maternal IV NAC 100 mg/kg every 6 h until delivery; neonatal IV NAC 12.5–25 mg/kg every 12 h × 5 doses	Placebo saline	Cerebrovascular coupling, inflammatory biomarkers	NAC preserved cerebrovascular coupling, increased IL-1Ra, reduced VEGF, and showed no major adverse events.
Wiest et al. (7)	Prospective PK cohort with randomised elements	Single centre (USA)	11 mothers, 12 infants with chorioamnionitis	Maternal IV NAC 100 mg/kg every 6 h; neonatal IV NAC 12.5–25 mg/kg every 12 h × 5 doses	Placebo saline	Maternal and neonatal pharmacokinetics	Demonstrated rapid placental transfer and gestational age–dependent neonatal clearance.
Buhimschi et al. (10)	Quadruple-blind RCT	Single centre (USA)	67 mothers and infants with Triple I	FDA acetaminophen protocol plus neonatal IV NAC	Placebo (5% dextrose)	Composite severe neonatal morbidity	Severe neonatal morbidity decreased from 47% to 21%; BPD reduced from 32% to 3%.
Jenkins et al. (11)	Prospective cohort extension	Single centre (USA)	16 infants from the original RCT	Follow-up of antenatal and neonatal NAC exposure	Placebo cohort	Long-term neurodevelopment	No cerebral palsy, autism spectrum disorder, or major developmental delay at 4 years.
Wiest PK et al. (7)	Neonatal PK sub-study	Single centre (USA)	12 infants receiving IV NAC	Neonatal IV NAC 12.5–25 mg/kg every 12 h × 5 doses	Placebo	Neonatal clearance and half-life	Preterm infants demonstrated prolonged NAC half-life and lower clearance.
Chang et al. (12)	Experimental animal study	Murine LPS model	CD-1 mice	IP NAC 1 g/kg before and after LPS	Saline	Cytokines, myelin markers	NAC prevented inflammation-induced preterm birth and preserved myelin integrity.
Buhimschi et al. (4)	Experimental animal study	Murine preterm labour model	C57Bl/6 mice	Oral NAC 1 g/kg before LPS	Oral saline	Delivery latency, glutathione levels	NAC prolonged latency to delivery and restored glutathione levels.
Sharabi et al. (13)	Experimental animal study	Rat neuroinflammation model	Pregnant rats	IV NAC 150 mg/kg post-LPS	IV saline	MRI/DTI biomarkers	NAC normalised diffusivity metrics and reduced white matter injury.
Gutzeit et al. (14)	Experimental animal study	Rat hypoxia model	Wistar rats	Antenatal and postnatal NAC	No NAC	NF-*κ*B, apoptosis, behaviour	NAC reduced inflammatory signalling and improved sensorimotor outcomes.
Zmora et al. (15)	Experimental animal study	Rat NEC inflammatory model	Sprague-Dawley rat pups	Antenatal and postnatal NAC	No NAC	NEC mortality, cytokines	NAC reduced NEC mortality, inflammatory cytokines, and caspase-3 activation.
Probyn, et al. (16)	Controlled four-group *in vivo* preclinical experiment with repeated physiological measurements over 5 consecutive treatment days.	In-utero preterm ovine model of endotoxin-induced fetal inflammation; fetuses were chronically catheterised via the femoral artery and vein, with an amniotic catheter.	21 Border Leicester x Merino fetal sheep. Treatment began at 95 days of gestation (term 147 days; about 0.67 of term; estimated weight about 1 kg). Groups: saline *n* = 6; LPS *n* = 6; LPS + NAC *n* = 6; NAC *n* = 3.	LPS + NAC: fetal IV LPS 1 mcg/kg bolus, followed immediately by direct fetal IV NAC 50, 100, or 200 mg over 5 h (*n* = 2 per dose), once daily for 5 days.	Primary comparison: LPS alone (1 mcg/kg fetal IV daily for 5 days). Additional controls: saline alone and NAC alone.	No single primary endpoint was explicitly specified. Assessed outcomes included fetal SaO2, PaO2, PaCO2, arterial pH, mean arterial pressure, heart rate, hematocrit, haemoglobin, glucose, lactate, plasma TNF-alpha, and fetal body weight.	Compared with LPS alone, LPS + NAC caused: − Greater SaO2 fall: 30.5 ± 3.3% vs. 19.0 ± 3.1% over 2 days (*p* = 0.027), and 27.2 ± 3.3% vs. 18.6 ± 1.7% over 3 days (*p* = 0.01). − Greater PaO2 fall: 6.7 ± 0.6 vs. 3.6 ± 0.7 mmHg over 2 days (*p* = 0.05), and 5.9 ± 0.7 vs. 3.7 ± 0.2 mmHg over 3 days (*p* = 0.04). − Greater Hct and Hb increases over 2 days (*p* = 0.002 and *p* = 0.001) and a greater MAP fall over 3 days (*p* = 0.05). − NAC alone produced no significant physiological change and did not significantly reduce TNF-alpha. The authors concluded that NAC exacerbated LPS-induced fetal hypoxemia and hypotension and induced polycythemia.
				NAC-only: saline bolus followed by NAC 100 mg (*n* = 2) or 200 mg (*n* = 1) over 5 h daily for 5 days.			

The translational animal studies employed murine and rat models of intrauterine inflammation, maternal hypoxia, preterm labour, and necrotising enterocolitis (NEC)-associated inflammatory injury. NAC administration protocols varied across studies but consistently targeted inflammatory and oxidative stress pathways. Key outcomes included inflammatory cytokines, glutathione depletion, apoptosis, MRI and diffusion tensor imaging biomarkers, white matter injury, NEC mortality, and sensorimotor performance.

#### Outcomes from human clinical studies

##### Neuroprotective and neurodevelopmental outcomes

Human studies demonstrated potentially favourable neuroprotective effects of N-acetylcysteine (NAC) in pregnancies complicated by maternal chorioamnionitis or intra-amniotic infection/inflammation (Triple I). Jenkins et al. (11) reported preservation of cerebrovascular coupling assessed using near-infrared spectroscopy coherence metrics following maternal and neonatal NAC administration. NAC exposure was also associated with improved inflammatory profiles, including increased interleukin-1 receptor antagonist (IL-1Ra) and reduced vascular endothelial growth factor (VEGF) concentrations (Table 2). Long-term follow-up findings from the Jenkins cohort extension demonstrated no major neurodevelopmental safety concerns associated with antenatal and neonatal NAC exposure at 4 years corrected age Jenkins et al. (11),. At 4 years corrected age, no cases of cerebral palsy, autism spectrum disorder, or significant developmental delay were observed among NAC-exposed children, although interpretation was limited by small sample size and limited statistical power (Table 2).

##### Severe neonatal morbidity and respiratory outcomes

Buhimschi et al. demonstrated the most clinically significant neonatal findings among the included human studies. In this quadruple-blind randomised controlled trial involving pregnancies complicated by Triple I, NAC administration was associated with substantial reductions in severe composite neonatal morbidity, decreasing from 47% in the placebo group to 21% in the NAC group (RR 0.45). Bronchopulmonary dysplasia (BPD) rates were also markedly reduced (32% vs. 3%), alongside a reduced need for intensive delivery-room resuscitation (Table 2). Collectively, these findings suggest that NAC may contribute to improved neonatal physiological adaptation and reduced inflammation-associated respiratory morbidity in high-risk preterm infants exposed to intrauterine inflammation.

##### Pharmacokinetic findings

Pharmacokinetic studies demonstrated rapid placental transfer of NAC and gestational age–dependent neonatal elimination patterns. Wiest et al. reported a cord-to-maternal concentration ratio of 1.4 ± 0.8, confirming effective fetal exposure following maternal intravenous administration. Maternal elimination half-life was approximately 1.2 ± 0.2 h, whereas neonatal half-life was prolonged in preterm infants compared with near-term infants (7.5 vs. 5.1 h), reflecting reduced clearance in premature neonates (Table 2). The neonatal pharmacokinetic substudy by Wiest et al. further demonstrated that gestational age substantially influenced NAC disposition, supporting gestational age–adjusted neonatal dosing strategies Wiest et al. (7).

#### Translational evidence from preclinical animal studies

Six translational animal studies evaluated the mechanistic and neuroprotective effects of N-acetylcysteine (NAC) in experimental models of intrauterine inflammation, hypoxia, preterm labour, and necrotising enterocolitis (NEC)-associated inflammatory injury (Table 2). Across studies, NAC consistently demonstrated anti-inflammatory, antioxidant, and anti-apoptotic effects.

Experimental murine models of lipopolysaccharide (LPS)-induced intrauterine inflammation demonstrated that NAC reduced pro-inflammatory cytokines, prolonged latency to delivery, restored fetal glutathione levels, and preserved myelin-related proteins and white matter integrity (4, 12). NAC administration also prolonged latency to delivery and restored maternal and fetal glutathione levels, suggesting attenuation of oxidative stress–mediated fetal injury (Table 2).

Neuroimaging findings from rat models further demonstrated that NAC normalised diffusion tensor imaging metrics associated with white matter injury following prenatal inflammatory exposure. Maternal NAC treatment following maternal inflammation significantly influenced brain micro-structure integrity as demonstrated by MRI-DTI scans. Additional studies reported reduced NF-*κ*B activation, decreased neuronal apoptosis, and improved sensorimotor performance following antenatal and postnatal NAC administration.

In NEC-associated inflammatory injury models, NAC exposure was associated with lower mortality, reduced inflammatory cytokine expression, and decreased caspase-3 activation, indicating potential protective effects against inflammation-mediated gastrointestinal and systemic injury. Collectively, these translational findings support the biological plausibility of NAC-mediated neuroprotection in maternal chorioamnionitis and Triple I through modulation of oxidative stress, inflammatory signalling, apoptosis, and white matter injury pathways (Table 2).

### Synthesis of evidence

#### Cerebrovascular coupling and autoregulation

Jenkins (11) reported that NAC maintained cerebrovascular coupling, a surrogate measure of cerebrovascular autoregulation, in the NAC group, while this coupling was dysregulated in placebo-exposed infants. There was no between-group difference in Doppler velocimetry or cerebral oxygenation in the NAC group.

#### Severe morbidity and respiratory outcomes

In the Triple I RCT (10), found large, significant decreases in rates of composite severe neonatal morbidity (21% vs. 47%) and BPD (3% vs. 32%, *p* = 0.006, RR = 0.1, 95% CI 0.01-0.73). The effect size was consistent across strata of GA, birth weight, sex, and race. NAC-exposed infants also required less resuscitation in the delivery room.

#### Cytokines and growth factors

Jenkins study (11) also found significant trajectories of higher IL-1 receptor antagonist and lower VEGF in the NAC group compared to placebo. In a mouse model of intrauterine inflammation, NAC consistently decreased IL-1β, IL-6, TNF-α, NF-*κ*B activity, and nNOS expression (12, 14, 15), which would be expected to abrogate or attenuate downstream tissue injury.

#### MRI/DTI

Sharabi et al. (13) found that when NAC was given 30 min after an LPS challenge in rats, mean, axial, and radial diffusivity were not different from controls in white matter or grey matter, while placebo-exposed animals had increased diffusivity, suggesting loss of microstructural integrity. Maternal NAC treatment following maternal inflammation significantly influenced brain micro-structure integrity as demonstrated by MRI-DTI scans. Limited human MRI data from Jenkins et al. (2016) found no adverse changes in MRI or MRS in NAC-exposed infants at term-equivalent age, supporting safety.

#### Neurodevelopment

Four-year follow-up data (11) from the Jenkins cohort showed that no infants in the NAC group were diagnosed with cerebral palsy or autism, while the numbers in the placebo group were small. While these data cannot rule out later neurodevelopmental injury, they are reassuring against delayed neurotoxic effects and raise the possibility of benefit, particularly for longer-term outcomes such as learning disorders.

#### Gastrointestinal

Zmora et al. (15) found that combined antenatal and postnatal NAC significantly decreased NEC-associated mortality and also decreased markers of ileal injury and inflammation. While NEC was not a pre-specified human outcome, these findings suggest potential protective effects in other organs in a highly inflammatory preterm phenotype.

#### Latency and survival

In the mouse model of LPS-induced preterm labour, NAC doubled the latency to delivery and reduced the incidence of stillbirth (4).

#### Maternal side effects

Human studies found no NAC-related maternal deaths or severe side effects. Infusion reactions were modest and did not need cessation. In one experiment, NAC did not affect maternal heart rate or blood pressure. NO moms reported anaphylactoid reactions, coagulopathies, or hepatic/renal damage in human studies. In Triple I, NAC did not increase cesarean delivery or postpartum haemorrhage. Infants and pregnant women remove NAC quicker (7), proposing a q6h dose or continuous infusion to maintain therapeutic levels. In LPS-induced preterm delivery mice, NAC normalised maternal and fetal hepatic glutathione (4).

#### Oxidative stress modulation and neuroprotection in neonatal HIE

A phase IIb clinical trial of moderate–to–severe HIE in neonates treated with therapeutic hypothermia found that intravenous NAC and calcitriol increased antioxidant capacity (17). Infants receiving NAC showed significantly higher GSH levels (*p* < 0.05) and a GSH/GSSG redox ratio increase of ∼2- to 3-fold relative to predicted oxidative stress injury. Additionally, NAC treatment significantly reduced oxidative damage markers (systemic and central nervous system) in newborns, indicating enhanced redox homeostasis during the acute phase of neonatal brain injury (*p* < 0.05). MRI brain damage was less severe in NAC-treated newborns than in hypothermia-treated infants.

#### Reduced neuroinflammation and motor damage in prenatal inflammation models

A preclinical study of prenatal inflammation–induced cerebral palsy (18) showed that postnatal systemic NAC therapy lowered neuroinflammatory activity in neonatal rabbits. NAC reduced microglial activation by 35%–40% in the experimental group. NAC therapy significantly reduced pro-inflammatory cytokines such as TNF-α and IL-1β (*p* < 0.01) compared to saline controls. NAC-treated rabbits had better motor function and fewer neurological impairments. These findings suggest NAC may prevent neuronal damage from inflammation.

#### Individual study details (RCTs, human)

NAC-treated infants maintained cerebrovascular coupling, had higher IL-1 receptor antagonist and lower VEGF trajectories, and had no adverse events. Wiest et al. (7), was a PK-focused RCT of pregnant people. Maternal NAC clearance was significantly accelerated in pregnancy (t½ = 1.2 h), and the cord: maternal ratio averaged 1.4, confirming rapid placental transfer. Neonatal PK differed by fetal maturity, with preterm infants having longer half-lives and more prolonged exposures than near-term infants. This trial informed dosing interval decisions. Buhimschi et al. (10), was a quadruple-blind RCT of 67 moms with amniocentesis-confirmed Triple I. The researchers gave NAC at 100 mg/kg IV administered at 1 g/200 mL in water over 15 min every 6 h until delivery, then the infant received 12.5–25 mg/kg every 12 h for five doses. In the FDA-approved acetaminophen overdose infusion treatment, moms and newborns received NAC. NAC significantly reduced composite severe morbidity (21% vs. 47%) and BPD (3% vs. 32%). Placental HDAC2 expression dropped, and white blood cell count and C-reactive protein decreased in NAC-treated infants, improving delivery room stability. There were no sex, race, GA, or birth weight differences. Table 3 shows Summary of included human studies.

**Table 3:** Summary of included human studies.

Author (Year)	Design	Setting	Population	GA (wk.)	Chorioamnionitis Definition	NAC Regimen	Comparator	Primary Outcomes	Followup	RR (CI)	*P*-value	Key Findings (with statistics)
Jenkins et al. (11)	Double-blind RCT	Single centre (USA)	22 mothers, 24 infants with clinical chorioamnionitis	>24	Clinical: maternal fever + ≥2 criteria (tachycardia, leukocytosis, uterine tenderness, foul fluid)	Antenatal IV 100 mg/kg q6h until delivery; neonatal IV 12.5–25 mg/kg q12h × 5 doses	Placebo saline	Cerebrovascular coupling (NIRS coherence)	Hospital discharge; 4-year follow-up cohort		*P* = 0.026	NAC preserved cerebrovascular coupling and improved inflammatory profile (↑ IL-1Ra, ↓ VEGF). No NAC-related adverse events. Normal MRI/MRS and no cerebral palsy or autism at 4-year follow-up.
Wiest et al. (7)	Prospective PK cohort with randomised elements	Single centre (USA)	11 mothers, 12 infants with chorioamnionitis	24–34 (preterm) and >34 (near-term)	Clinical: maternal fever + ≥2 criteria (tachycardia, leukocytosis, uterine tenderness, foul fluid)	Maternal IV 100 mg/kg q6h; neonatal IV 12.5–25 mg/kg q12h × 5 doses	Saline (randomised allocation to NAC vs. placebo)	Maternal and neonatal PK parameters (clearance, t½, Vd, cord: maternal ratio)	Through 5 postnatal doses		*P* = 0.03	Maternal half-life 1.2 ± 0.2 h. Cord: maternal concentration ratio 1.4 ± 0.8, confirming placental transfer. Neonatal half-life 5.1 h (near-term) vs. 7.5 (preterm).
Buhimschi et al. (10)	Quadruple-blind RCT	Single centre (USA)	67 mothers, 67 infants with Triple I	24–34	Triple I: amniocentesis-confirmed intra-amniotic infection/inflammation (IL-6 ≥ 2.6 ng/mL, positive Gram stain, or positive culture)	Intrapartum NAC: FDA regimen (loading dose 150 mg/kg over 1 h, then 50 mg/kg over 4 h, then 100 mg/kg over 16 h); neonatal IV 12.5–25 mg/kg q12h × 5 doses	Placebo (5% dextrose)	Composite severe neonatal morbidity (death, severe IVH, PVL, NEC ≥ II, severe BPD)	Hospital discharge	RR 0.45;95% (CI) 0.21–0.95	*P* = 0.037	Severe neonatal morbidity 21% vs. 47% (RR 0.45). BPD 3% vs. 32%. Reduced need for intensive delivery room resuscitation and decreased placental HDAC2 expression.
Jenkins follow-up (11)	Prospective cohort extension	Single centre (USA)	16 infants (8 NAC, 8 placebo) from the original RCT	Same as RCT	Same as RCT	Same as RCT	Same as RCT	Neurodevelopmental outcomes (CP, ASD, developmental delay)	4 years corrected age		*P* = 0.026	Cerebral palsy: 0/8 NAC vs. 0/9 control.
												ASD: 1/12 NAC vs. 0/9 control (1 preterm infant with fine motor delay and ASD at 4 years).
												Developmental delay: 2/12 NAC (1 spastic quadriplegia with grade IV IVH/PVL; 1 fine motor delay + ASD) vs. 2/9 control (1 speech delay; 1 fine motor delay).
												No *p*-values, RR, or CI reported; sample too small for formal inferential testing.
												Interpretation: Preliminary reassurance against NAC neurotoxicity; insufficient power to establish efficacy for neurodevelopmental outcomes.
Wiest et al. (7)	Neonatal PK sub-study	Single centre (USA)	12 infants receiving IV NAC	Same as RCT	Same as RCT	Neonatal IV NAC 12.5–25 mg/kg q12 h × 5	Placebo	Neonatal clearance, t½	PK finding (not follow-up): clearance that changes with gestational age supports individualised dosing		*P* = 0.03	(t½): Preterm: 7.5h
												Near-term: 5.1 h
												Clearance:
												Lower preterm infants
												eliminate drugs more slowly.
												Large premature babies reflect bodily water. Interpretation:
												GA greatly impacts NAC pharmacokinetics.
												Supports weight- and GA-adjusted dosage over fixed.

### Individual preclinical study details

In a study conducted by Chang et al. (12),, the authors found that in a CD-1 mouse intrauterine LPS model of preterm birth, NAC given at 1 g/kg IP around the time of LPS exposure was able to prevent preterm birth from 79% to near zero. It also lowered fetal brain IL-1β/IL-6/TNF-α. In addition, NAC reduced loss of neurofilament (NF-H) and myelin (MBP, PLP) markers, suggesting structural neuroprotection. In another study by Buhimschi et al. (4),, Oral NAC (1 g/kg) given to LPS-challenged C57Bl/6 mice resulted in doubling of latency to delivery and improved fetal survival, while restoring maternal and fetal hepatic glutathione levels. This directly supports a redox mechanism of action. Sharabi et al. (13), showed that in a rat model of LPS exposure, NAC given 30 min after the onset of inflammation normalised DTI diffusivity measures (mean, axial, radial) in offspring brains at PND 25. Pregnant Sprague–Dawley dams (*n* = 6) at day 18 of gestation received either an intraperitoneal injection of LPS or saline (Control) at time 0. Animals were randomised to receive an intravenous injection (tail vein) of NAC or saline at time +30 min. Pups were delivered spontaneously and allowed to mature until postnatal day 25. Male offspring (6–8 per group) were examined by MRI and analysed using voxel-based analysis. The offspring of NAC-treated LPS PS dams demonstrated reduced mean, axial and RD levels in most regions, similar to the saline group. Maternal NAC treatment following maternal inflammation significantly influenced brain micro-structure integrity as demonstrated by MRI-DTI scans (13). In the study conducted by Gutzeit et al. (14),, maternal antenatal or offspring postnatal NAC in a hypoxia model resulted in decreased NF-*κ*B, nNOS, TNF-α, and IL-6 protein expression, decreased apoptosis by TUNEL staining, and improved righting reflex performance. These changes were related to performance on functional outcomes. Zmora et al. (15), found that in an NEC model, maternal antenatal and offspring postnatal NAC resulted in decreased mortality and decreased ileal cytokines and caspase-3. The benefit was the greatest with the combined antenatal and postnatal NAC exposure, suggesting a dose-dependent or cumulative effect across perinatal periods. On the other hand, Probyn et al. (16), concluded that, although NAC alone is without adverse physiological effects on the fetus, when used in the presence of the inflammatory agent LPS, NAC exacerbates the deleterious physiological effects of LPS. Their findings therefore suggested that NAC may not be a suitable neuroprotective agent when administered antenatally in a clinical setting to a fetus with evidence of inflammation such as chorioamnionitis (16). Table 4 shows summary of included preclinical animal studies.

**Table 4:** Summary of included preclinical animal studies.

Author (Year)	Animal Model	Species	Inflammatory Stimulus	Population	NAC Regimen	Comparator	Key Outcomes Assessed	Timeline of Evaluation	RR (CI)	*P*-value	Key Findings (with statistics)
Chang et al. (12)	Intrauterine LPS-induced preterm birth and brain injury	CD-1 mice	Intrauterine LPS (E. coli 055: B5, 25 μg/uterus) at E15	Dams: 20–25/group; offspring followed to P30	IP NAC 1 g/kg 30 min before LPS and 4 h after LPS	Saline (same volume, same timing)	PTB rate, fetal brain cytokines (IL-1β, IL-6, TNF-α), myelin markers (NF-H, MBP, PLP)	24 h post-LPS (cytokines); P30 (myelin)	RR 0.45 (95% CI 0.26–0.83),	*P* = 0.008	NAC prevented inflammation-induced preterm birth from 79% to 38% and significantly reduced IL-1β, IL-6, and TNF-α levels. Myelin markers are preserved in the fetal brain.
Buhimschi et al. (4)	Maternal LPS-induced preterm labor	C57Bl/6 mice	IP LPS (E. coli, 50 μg/mouse) at E15–17	Dams: 10–12/group	Oral NAC 1 g/kg 30 min before LPS	Oral saline (same volume, same timing)	Latency to delivery, fetal viability, maternal/fetal hepatic GSH levels	Up to 48 h post-LPS	LPS was followed by a reduction in maternal [LPS: 26.3 nmol/mg [95% CI 19.9–32.8] vs. saline solution (CRL): 41.3 nmol/mg [95% CI 34.7–47.9, *P* < .01]] and fetal GSH [LPS: 19.7 nmol/mg [95% CI 11.7–27.8] vs. CRL: 34.5 nmol/mg [95% CI 32.0–37.0, *P* < .001]]. This decline was reversed by NAC [NAC/LPS maternal GSH: 37.0 nmol/mg [95% CI 22.5–51.5] and fetal GSH: 28.4 nmol/mg [95% CI 22.8–33.9]].	*P* < 0.01	NAC doubled latency to delivery, improved fetal survival, and restored maternal and fetal glutathione levels, indicating reduced oxidative stress.
Sharabi et al. (13)	Maternal LPS-induced neuroinflammation	Pregnant rats (strain not specified)	IP LPS (E. coli, 200 *μ*g/kg) at E18	Dams: 6/group; male offspring at P25: 6–8/group	IV NAC 150 mg/kg 30 min post-LPS	IV saline (same volume, same timing)	MRI/DTI metrics (mean, axial, radial diffusivity) in the offspring brain at P25	P25		*P* < 0.05	NAC normalised mean, axial, and radial diffusivity values, indicating protection against white-matter injury caused by prenatal inflammation.
Gutzeit et al. (14)	Maternal hypoxia-induced brain injury	Pregnant rats (Wistar)	Maternal hypoxia (7% O_2_, 60 min) at E19	66 pups across 4 groups: control, hypoxia, antenatal NAC, postnatal NAC	Antenatal: IP NAC 500 mg/kg 30 min pre-hypoxia; Postnatal: IP NAC 500 mg/kg/day ×3 days	No NAC (saline)	NF-κB, nNOS, TNF-α, IL-6 protein expression; TUNEL apoptosis; righting reflex	24 h post-hypoxia (proteins); P1–3 (behavior)		*P* < 0.05	NAC significantly reduced NF-κB activation, TNF-α, IL-6, and neuronal apoptosis while improving sensorimotor righting reflex performance compared with untreated animals.
Zmora et al. (15)	NEC model (combination of formula feeding, asphyxia, LPS)	Rat pups (Sprague-Dawley)	NEC induction: formula feeding + asphyxia (100% N₂, 90 s) + LPS (4 mg/kg) × 4 days	168 pups across 5 groups	Antenatal: NAC in drinking water (1 g/L) from E14; Postnatal: IP NAC 500 mg/kg/day ×3 days	No NAC	NEC mortality, ileal histologic injury, ileal cytokines (TNF-α, IL-1β, IL-6), caspase-3 activity	Day 4 of the NEC protocol		*P* < 0.05	NEC mortality was reduced to 11% vs. 34% in the untreated NEC group. Combined antenatal + postnatal NAC produced the largest reduction in inflammatory cytokines and caspase-3 activation.
Probyn, et al. (16)	In utero, chronically catheterised preterm fetal model of endotoxin-induced inflammation	Ovine (Border Leicester x Merino sheep)	Direct fetal IV LPS, 1 microgram/kg estimated fetal weight, as a bolus once daily for 5 days	21 catheterized fetuses at 95 days gestation (∼0.67 term; human-equivalent 24–26 weeks): saline *n* = 6; LPS *n* = 6; LPS + NAC *n* = 6; NAC *n* = 3	Direct fetal IV NAC for 5 h immediately after each LPS dose: 50, 100, or 200 mg (*n* = 2 per dose), daily for 5 days. NAC-only: 100 mg (*n* = 2) or 200 mg (*n* = 1)	Primary: LPS alone (*n* = 6). Additional controls: saline (*n* = 6) and NAC alone (*n* = 3)	Fetal SaO2, PaO2, PaCO2, arterial pH, MAP, HR, Hct, Hb, glucose, lactate, plasma TNF-alpha, and fetal body weight	Treatment at 95–99 days of gestation. MAP/HR recorded before, during, and after each 5-h infusion; blood sampled before dosing and hourly for 6 h; TNF-alpha at 0, 2, and 6 h. Maximal 3–6-h changes analyzed over days 1–2 and 1–3; euthanasia at 105 days		Post-hoc:	Compared with LPS alone, LPS + NAC caused larger falls in SaO2: 30.5 ± 3.3% vs. 19.0 ± 3.1% over 2 days (*p* = 0.027) and 27.2 ± 3.3% vs. 18.6 ± 1.7% over 3 days (*p* = 0.016). PaO2 also fell more: 6.7 ± 0.6 vs. 3.6 ± 0.7 mm Hg (*p* = 0.050) and 5.9 ± 0.7 vs. 3.7 ± 0.2 mm Hg (*p* = 0.048). Over 2 days, Hct rose 7.4 ± 0.6% vs. 5.1 ± 1.1% (*p* = 0.002) and Hb rose 2.5 ± 0.2 vs. 1.7 ± 0.4 g/dL (*p* = 0.001). Over 3 days, MAP fell 6.6 ± 1.2 vs. 2.2 ± 2.0 mm Hg (*p* = 0.050). NAC alone caused no significant physiological change. The authors concluded that NAC exacerbated LPS-induced hypoxemia, hypotension, and polycythemia
										SaO2: 0.027 (2 d), 0.016 (3 d)	
										PaO2: 0.050, 0.048	
										Hct: 0.002 (2 d)	
										Hb: 0.001 (2 d)	
										MAP: 0.050 (3 d)	
										pHa 0.056; lactate 0.065; HR 0.077/0.070 (trends)	
										PaCO2, glucose, TNF-alpha: NS	

### Pharmacokinetics and exposure

The Wiest trial (7) demonstrated faster maternal NAC clearance during pregnancy (t½∼1.2 h), supporting a q6h dose to prevent subtherapeutic trough levels. The maternal-to-cord blood ratio averaged 1.4, indicating rapid placental transfer. Because neonatal half-life was prolonged and gestation-dependent (7.5 h in preterm babies vs. 5.1 h in near-term infants), the postnatal neonatal dosage interval should be lengthened to minimise accumulation. PK properties allow weight-based dosing and infusion technique selection (e.g., FDA-approved Triple I RCT acetaminophen overdose protocol).

Regarding safety, human studies showed no significant adverse effects or deaths. NAC did not affect hemodynamic stability in one trial. No infusion-related mild responses were noted. Animal investigations showed no NAC-related mortality; inflammatory models increased survival (4, 15). This strong safety profile and decades of clinical usage in acetaminophen overdose suggest practicality and acceptability in obstetric populations.

The Cochrane Risk of Bias 2 (RoB 2) tool checked randomised controlled trials for bias (7, 10, 11), ROBINS-I for non-randomised and secondary analyses (7, 18), and SYRCLE for animal studies (4, 12–15). The randomised studies had negligible bias, but limited sample sizes and statistical power raised concerns. The non-randomised follow-up and pharmacokinetic subset studies had a moderate risk of bias due to confounding factors, selection bias, poor data management, and selective reporting. Preclinical investigations showed a low risk, but inadequate reporting of randomisation and blinding protocols raised concerns. The evidence base is stronger since biological plausibility is consistent, but it is poorer because clinical sample sizes are small and outcomes are surrogate-based.

## Discussion

### Principal findings of human clinical studies

In chorioamnionitis (Triple I), prenatal or intrapartum NAC may minimise inflammation-mediated newborn morbidity. In safe human clinical trials, cerebrovascular control, severe morbidity, BPD, and cytokine and protein modulation improved.

Drug absorption and prenatal exposure were predicted by pharmacokinetics. Mechanisms were revealed by reproducible brain, lung, and gut injury improvements in animal models. Interestingly, human clinical trials, pharmacokinetic research, and animal studies give equal results (6–8, 11).

In the Triple I RCT, which included 67 participants, the BPD rate was reduced from 32% to 3%, which was a notable neonatal neuroprotection therapy success in recent decades. However, this result is derived from modest samples at a single centre and has wide confidence intervals (BPD reduction: 3% vs. 32%, *p* = 0.006, RR = 0.1, 95% CI 0.01-0.73). Primary Composite morbidity score ≥2: RR = 0.45, 95% CI = 0.213–0.952, *p* = 0.037 (10).

Lung and brain traumas may share oxidative stress, inflammation, and endothelial dysfunction due to the magnitude of the effect. Jenkins suggests that NAC modulates cerebral autoregulation, which alters white matter perfusion in systemic inflammation, prolonging cerebrovascular coupling (11). The human double-blind RCT by Jenkins et al. of 22 mothers and 24 infants with clinical chorioamnionitis did not report relative risks or confidence intervals, as the trial was powered for safety and not efficacy. Cerebrovascular coupling was preserved in NAC-treated infants and abolished in saline controls. In the NAC group, there was a significant correlation between CBF velocities in the MCA and velocities in the ACA (*p* = 0.0003) and BA (*p* = 0.003), but not in controls. These correlations were significant after Bonferroni correction (*p* < 0.0125). Infants treated with NAC had significantly more of the anti-inflammatory IL-1Ra and less of the pro-inflammatory VEGF over time compared with controls (*p* ≤ 0.014). Preterm infants had higher basal ganglia mI/NAA ratios in controls (1.4 ± 0.23) than NAC-treated infants (0.92 ± 0.30; *p* = 0.026). No adverse events related to NAC were reported in mothers or babies. Twenty-one chorioamnionitis-exposed infants were available for follow-up to 3−4 years of age (9 controls, 12 NAC-treated infants). Two of the 9 control infants had developmental delays. Two of 12 infants treated with NAC had developmental delays. There were no cases of cerebral palsy or autism spectrum disorder in either group, and no statistically significant difference in developmental delay was observed between NAC and placebo-treated infants in the 4-year neurodevelopmental follow-up of the human RCT cohort (*n* = 16 infants; 8 NAC, 8 placebo). Because of the small sample size, formal inferential testing was not performed, and so no *p*-values, relative risks or confidence intervals were calculated or reported. These data offer preliminary reassurance against long-term neurotoxicity of NAC but do not confirm efficacy for neurodevelopmental outcomes (11).

Wiest et al. (7) conducted a prospective pharmacokinetic study of 11 mothers and 12 infants receiving IV NAC for chorioamnionitis; relative risks and confidence intervals were not reported, as the study was only meant to characterise NAC disposition. Maternal clearance was significantly faster than in nonpregnant adults (mean t½ 1.2 ± 0.2 h vs. ≈6 h in nonpregnant adults). The mean NAC cord to maternal concentration ratio was 1.4 ± 0.8, and cord NAC concentration was significantly correlated with time from end of maternal infusion (*p* = 0.0005, R2 = 0.88). The neonatal t½ was significantly shorter in near-term infants compared with preterm infants (5.1 ± 1.3 h vs. 7.5 ± 2.0 h; *p* = 0.02). Clearance was significantly greater in near-term infants (53.7 ± 11.3 vs. 45.0 ± 8.2 mL/h/kg; *p* = 0.021). Mothers who delivered prematurely had a higher maternal volume of distribution than those who delivered near-term (*p* = 0.03). No adverse effects were noted in any mother or infant during NAC administration (11).

### Principal findings of preclinical animal studies

In the preclinical animal study Chang et al. (12),, pregnant CD-1 mice (*n* = 3 saline control, *n* = 24 LPS, *n* = 16 NAC + LPS) received an intrauterine LPS injection (100 *μ*g/mouse) on gestational day 15. This is the only animal study in your review that reports RR and CI formally. Intrauterine LPS caused preterm birth in 79% vs. 0% of controls (*p* < 0.05). NAC pretreatment (100 mg/kg IP) resulted in a significant decrease in the probability of preterm delivery: RR 0.45 (95% CI 0.26-–.83), *p* = 0.008, reducing the rate of preterm birth from 79% to 38%. NAC significantly reduced LPS-induced IL-6 expression in the uterine fundus (2,858.12 ± 750.79 vs. 30.38 ± 15.49 arbitrary units, *p* < 0.05) and lower uterine segment (212.90 ± 48.60 vs. 49.11 ± 8.81, *p* < 0.05) and IL-6 expression in the placenta (17.28 ± 1.89 vs. 10.9 ± 1.32, *p* < 0.05) in maternal tissues. There was no statistically significant (*p* > 0.05) reduction of TNF-α and IL-1β in the lower uterine segment. NAC caused large numerical reductions in IL-6, TNF-α and IL-1β in the fetal brain, but these were not statistically significant (*p* > 0.05), possibly due to small group sizes (*n* = 6–8). Structurally, LPS increased NF-H staining (marker of axonal injury) and decreased MBP and PLP staining (markers of myelination) at PND 1 and 30, respectively; all were attenuated by NAC (*p* < 0.05 by Kruskal–Wallis with Mann–Whitney pairwise comparisons). This is a preclinical mouse study, so no human RR/CI can be derived from it, but it is the only animal study in this review that provides a formally calculated RR with CI for a clinically relevant outcome (prevention of preterm birth) (12).

This seminal preclinical animal study (Buhimschi IA et al) involved pregnant C57Bl/6 mice (*n* = 12 per group) receiving intraperitoneal LPS (10 μg) or saline on gestational day 16 with or without oral NAC (1 g/kg). No RR or CI reported. Fisher's exact test and ANOVA were used to analyse the primary preterm birth outcome. NAC significantly increased latency to delivery: LPS group 16.8 h (95% CI 15.9–17.6) vs. NAC/LPS group 35.2 h (95% CI 21.0–49.2; *p* = 0.008 vs. LPS), compared with saline controls at 54.7 h (95% CI 43.8–65.5; *p* < 0.001 vs. LPS). NAC significantly reduced the rate of preterm delivery at 36 h post-injection (LPS vs. NAC/LPS, *p* = 0.037). Fetal Survival All LPS pups were stillborn, whereas 75% of NAC/LPS dams gave birth to live or mixed liveborn/stillborn pups (*χ*^2^ = 57.2, *p* < 0.001). Fetal mortality significantly decreased at 16 h. post-LPS in the NAC/LPS group (27% vs. 58%; *χ*^2^ = 17.2, *p* < 0.001). LPS significantly depleted maternal hepatic GSH [26.3 [95% CI 19.9–32.8] vs. control 41.3 nmol/mg [95% CI 34.7–47.9]; *p* < 0.01] and fetal hepatic GSH [19.7 [95% CI 11.7–27.8] vs. control 34.5 nmol/mg [95% CI 32.0–37.0]; *p* < 0.001] and both were restored by NAC [maternal 37.0 [95% CI 22.5–51.5]; fetal 28.4 nmol/mg [95% CI 22.8–33.9]]. Hepatic GSH in NAC/LPS mothers with living pups was significantly higher than in NAC/LPS mothers with pups that died *in utero* [45.9 [95% CI 21.8–63.6] vs. 19.2 nmol/mg [95% CI 16.7–28.1]; *p* = 0.012]. No formal calculations of RR or CI for preterm birth were performed, as this is a preclinical mouse study and no human clinical outcomes can be inferred from it (4).

In a preclinical animal study Sharabi et al. (13), pregnant Sprague–Dawley rats (*n* = 6 dams per group) received intraperitoneal LPS (500 μg/kg) or saline on gestational day 18, followed 30 min later by IV NAC (300 mg/kg) or saline. Male offspring (5–8/group) were assessed by MRI-DTI at postnatal day 25. No RR or CI reported—DTI analysis performed using voxel-based ANOVA (SPM software) with a significance threshold of *p* < 0.001; pup weight comparisons performed with *p* < 0.05. LPS-SAL pups weighed significantly less than controls (5.7 ± 0.6 vs. 6.2 ± 0.8 g; *p* < 0.05), while LPS-NAC pups (6.3 ± 0.4 g) were equal to controls. Litter size was significantly decreased in both LPS groups compared to controls (LPS-SAL: 4.7 ± 1.2 vs. LPS-NAC: 6.3 ± 1.75 vs. control: 12.4 ± 5.5 pups; *p* < 0.05), with no significant difference between LPS-SAL and LPS-NAC groups. LPS-SAL offspring had significant increases of mean diffusivity (MD) on DTI-MD maps in several white and grey matter regions [corpus callosum, external capsule, thalamus, hippocampus (CA1, CA3), fimbria, auditory cortex and hypothalamus] compared to controls (*p* < 0.015 across regions); MD values were normalized to near-control levels in the corpus callosum/external capsule, auditory cortex and mammillary body after NAC treatment (*p* < 0.05 between LPS-NAC and LPS-SAL). LPS-SAL offspring showed significantly increased radial diffusivity (RD) on DTI-RD maps in similar areas (*p* < 0.05 compared to controls); NAC significantly reduced RD to near-control levels in broad areas of the medial lemniscus, entorhinal cortex, corpus callosum, hippocampus, thalamus and fimbria (*p* < 0.05 between LPS-NAC and LPS-SAL). FA and RA values between the groups did not show significant differences. This is a preclinical rat-only study; no RR or CI were formally calculated, and no human clinical outcome can be derived from it (13).

In preclinical animal study Gutzeit et al. (14) 66 newborn Sprague–Dawley rat pups in 4 groups were exposed to a hypoxia protocol (5% O2, 95% N2, 10 min, three times daily for 5 days) with either antenatal maternal NAC (NAC-HYP, *n* = 23) or postnatal offspring NAC (HYP-NAC, *n* = 21) compared to hypoxia alone (HYP, *n* = 11) and controls (CON, *n* = 11). No RR or CI were reported. The study used one-way ANOVA with Holm-Sidak *post-hoc* pairwise comparisons (*p* < 0.05). Hypoxia significantly increased brain NF-*κ*B p65 and nNOS of offspring compared to controls (2.20 ± 0.08 vs. 0.53 ± 0.12; 1.99 ± 0.46 vs. 0.80 ± 0.08 U; *p* < 0.05) and both antenatal maternal NAC (NAC-HYP: 0.88 ± 0.06; 0.70 ± 0.25 U; *p* < 0.05) and postnatal offspring NAC (HYP-NAC: 0.96 ± 0.09; 1.03 ± 0.11 U; *p* < 0.05) significantly reduced these levels vs. hypoxia alone. Hypoxia significantly increased brain TNF-α and IL-6 vs. controls (1.63 ± 0.25 vs. 0.50 ± 0.01; 2.43 ± 0.14 vs. 0.90 ± 0.11 U; *p* < 0.05); both NAC regimens significantly reduced these levels vs. hypoxia (NAC-HYP: 0.71 ± 0.10; 0.53 ± 0.05; HYP-NAC: 0.95 ± 0.07; 0.85 ± 0.15 U; *p* < 0.05). Both NAC regimens reduced brain apoptosis by TUNEL grading from grade 2 (hypoxia) to grade 1 (controls) (*p* < 0.05). No differences in serum IL-6 were found between groups. Hypoxia caused sensorimotor dysfunction (righting reflex: 5.44 ± 4.2 vs. control 3.54 ± 2.01 s; *p* < 0.05); antenatal NAC attenuated this significantly (3.91 ± 2.77 vs. 5.44 ± 4.2 s; *p* < 0.05), while postnatal NAC did not reach statistical significance. Pups from NAC-treated dams were significantly heavier on day 5 compared with HYP pups (9.4 ± 0.74 vs. 8.88 ± 0.21 g; *p* < 0.05). Mortality trended lower in NAC groups (NAC-HYP 8.69%; HYP-NAC 4.76%) compared to HYP (20%) but did not reach statistical significance. There was a significant increase in maternal liver glutathione in NAC-treated dams compared to controls (52.84 ± 4.12 vs. 34.70 ± 3.56 pmol; *p* < 0.05). This is a preclinical model of hypoxia in rats—no RR or CI were formally calculated, and no human clinical outcomes can be extrapolated (14).

In another study, Sprague-Dawley rat pups (*n* = 168 in 5 groups) were subjected to NEC induction by formula feeding, asphyxia and LPS administration over 4 days in this preclinical rat NEC model study Zmora et al. (15). There was a significant reduction in NEC-associated mortality in the combined antenatal + postnatal NAC group compared to the untreated NEC group (11% vs. 34%; (no RR or CI were formally calculated and reported by the authors). The histological injury scores, pro-inflammatory cytokines (TNF-α, IL-1β, IL-6) and caspase-3 apoptotic activity in the ileum were significantly lower in the NAC-treated groups compared with the untreated NEC controls (*p* < 0.05 for all). Antenatal + postnatal NAC regimen resulted in the greatest reduction in inflammatory markers and apoptosis compared to antenatal-only or postnatal-only NAC. No relative risks or confidence intervals were reported formally since the authors used ANOVA-based group comparisons with *post-hoc* testing. This is an animal study only—no human data or clinical RR/CI can be obtained from this (15).

In animals, NAC improved sensory capacities, diffusion tensor imaging metrics, structural connections, apoptosis, and myelin basic protein loss.

Another noteworthy conclusion was no opportunity window limit. The prenatal-postnatal combination lowered Zmora NEC mortality and inflammatory injury signs in most (15). Sharabi found NAC effective 30 min after LPS injury (13). Chorioamnionitis is diagnosed when labour is 50% complete, and inflammation is fast (9). Clinically and in practice, NAC treatment before and after inflammatory damage is effective.

On the other hand, the ovine LPS model pre-clinical study by Probyn et al. (16)showed that in the presence of LPS, NAC compromises fetal physiological status, suggesting that it may not be a suitable antenatal treatment for a fetus with evidence of inflammation (16).

### Mechanistic implications

The neuroprotective effects of NAC are likely multifactorial. NAC replenishes intracellular glutathione stores and directly scavenges reactive oxygen species, thereby reducing oxidative stress–mediated cellular injury. Inflammatory activation during chorioamnionitis stimulates NF-*κ*B signalling and downstream cytokine release, including IL-1β, IL-6, and TNF-α, which contribute to oligodendrocyte injury, impaired cerebrovascular regulation, and white matter damage. NAC-mediated suppression of these inflammatory pathways observed in both human and animal studies suggests a potential mechanism for reducing neuroinflammation-associated injury (12, 14). Other experimental neuroinflammatory models have similarly demonstrated that targeted anti-inflammatory interventions may attenuate cerebral palsy–related inflammatory injury pathways (11).

Additional mechanistic observations included reduced placental HDAC2 expression and normalisation of diffusion tensor imaging metrics following NAC exposure, suggesting possible epigenetic and microstructural protective effects (10, 13). These findings are particularly relevant because pre-oligodendrocytes are highly vulnerable to inflammatory and oxidative injury during preterm brain development.

### Timing, dosing, and pharmacokinetic considerations

The data indicate that neutralising the cytokine surge within hours after chorioamnionitis diagnosis is preferable. However (13), found an effect after inflammatory insult, opening a treatment window after diagnosis. A dose of 100 mg/kg q6h IV infusions targets prenatal and intrapartum fetal NAC exposure with tolerable maternal antioxidant exposure. The FDA overdose infusion protocol proved practicable and practical in Triple I (10). To minimise buildup, postnatal dose should employ neonatal (12.5–25 mg/kg q12 h) and reflect newborns' gestationally increased half-life.

The included studies suggest that the timing of administration may be critical for therapeutic efficacy. Both prophylactic and post-inflammatory NAC administration demonstrated benefit in translational models, indicating a potentially broader therapeutic window than previously assumed (13, 15). This observation has important clinical implications because maternal chorioamnionitis is frequently diagnosed after inflammatory injury has already begun.

Pharmacokinetic studies demonstrated rapid placental transfer and gestational age–dependent neonatal clearance (7). Preterm neonates exhibited prolonged elimination half-life compared with near-term infants, supporting the need for gestational age–adjusted dosing strategies. Current evidence also suggests that combined maternal and neonatal administration may provide greater benefit than isolated exposure, although optimal dosing regimens remain uncertain.

The pharmacokinetic modelling in pregnancy supports q6h dosing based on the NAC maternal rapid clearance (t½∼1.2 h). This differs from continuous acetaminophen overdose procedures. The latter may increase tolerance and fetal exposure by maintaining a steady-state NAC concentration without peaks and troughs. Sharabi et al.'s (13) findings suggest that maternal NAC therapy may be effective in human pregnancies associated with maternal/fetal inflammation, such as preterm rupture of membranes and chorioamnionitis. This suggests that therapeutic (not just prophylactic) timing can rescue microstructural damage (13). The Buhimschi FDA regimen (loading dose and long-term maintenance) was a good compromise. Biomarker feedback (IL-6, oxidative stress markers) can guide dosage escalation in extreme prematurity or severe inflammation in future PK/PD investigations.

Postnatal NAC dose needs careful attention and individualisation. Preterm newborns have a 7.5-hour half-life for NAC clearance, while near-term infants have 5.1 h. Without an extended dosage interval, preterm infants risk buildup and toxicity. The (11) study's five postnatal doses may be a good starting point, although newborns with Funisitis or severe fetal inflammatory response may need a longer course. The oral NAC bioavailability in newborns is unclear. In the immediate postnatal period, intravenous administration is preferable.

**Figure 3:**
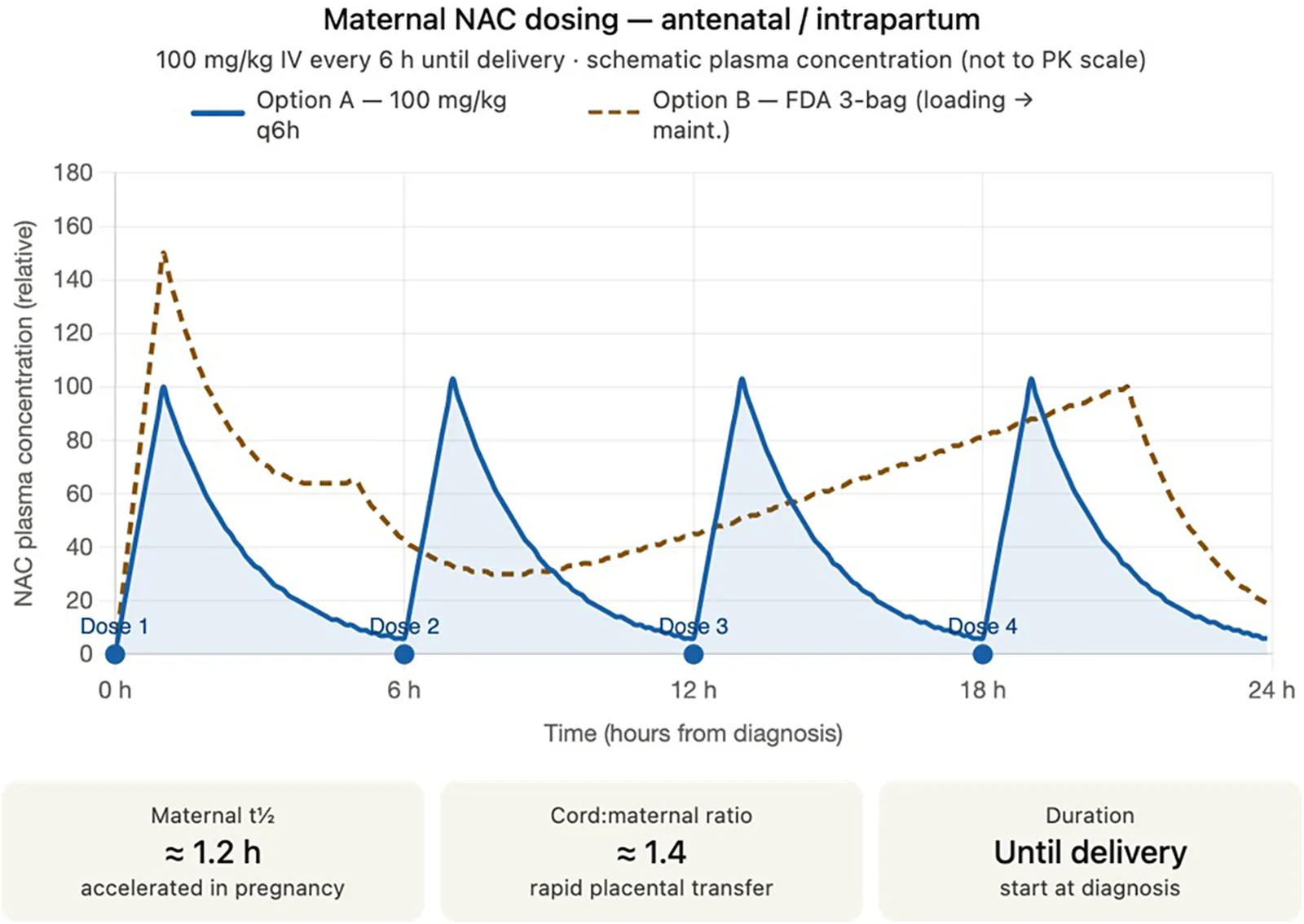
Maternal NAC dosing—antenatal/intrapartum.

**Figure 4:**
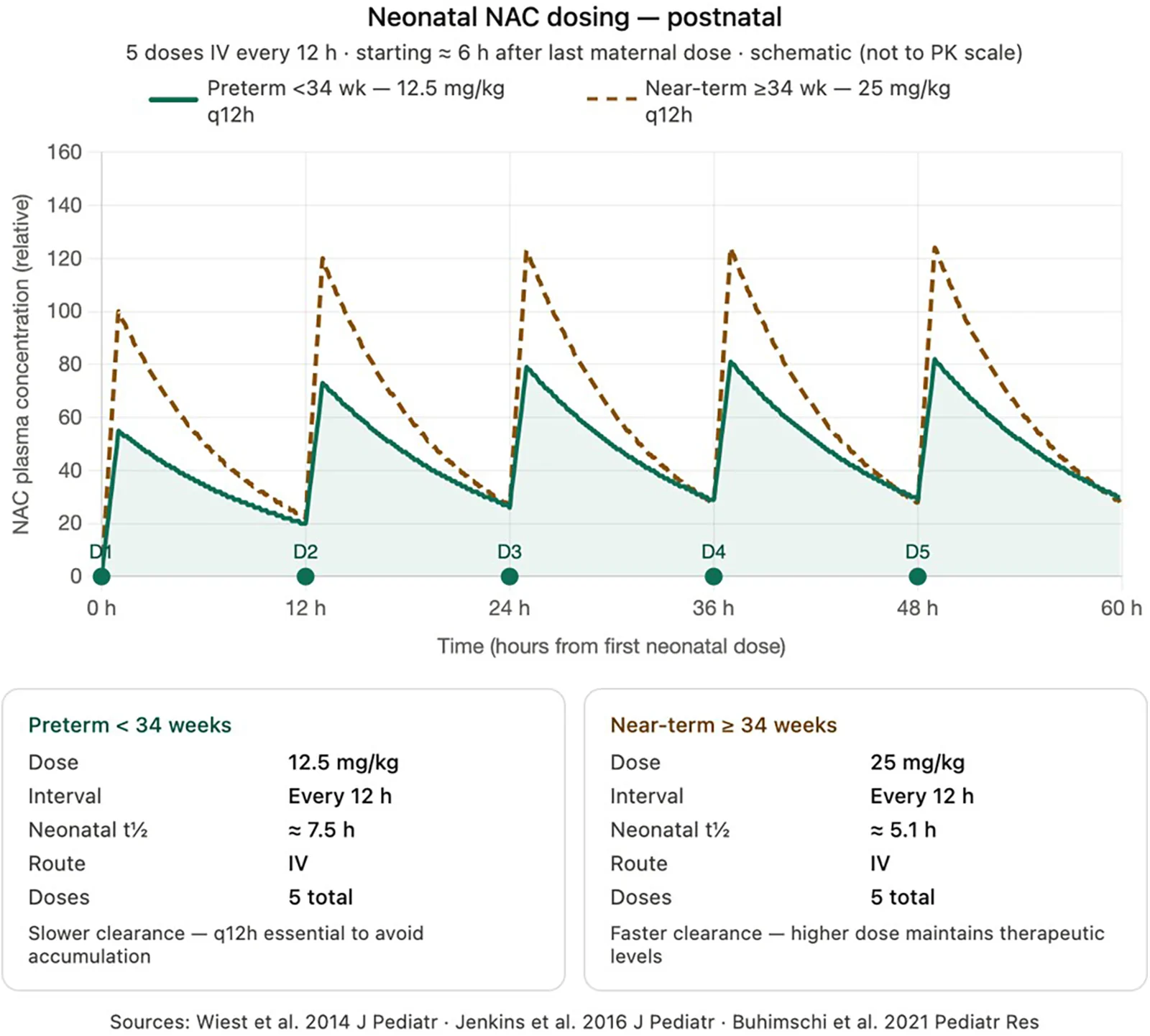
Neonatal NAC dosing—postnatal.

Figure 3 shows the maternal NAC Dosing—Antenatal/Intrapartum. Figure 4: Neonatal NAC Dosing—Postnatal. Table 5: NAC Dosing Reference—Chorioamnionitis/Triple I.

**Table 5:** NAC dosing reference—chorioamnionitis/triple I.

Domain	Subsection	Regimen/finding	Rationale/notes	Source
Clinical sequence	Overall workflow	Diagnosis: chorioamnionitis/Triple I. Maternal NAC is started antenatally or intrapartum and continued until delivery; after delivery, switch to the neonatal regimen.	Maternal phase: q6h dosing until delivery. Neonatal phase: q12 h dosing for 5 total postnatal doses.	
Maternal dosing	Option A—q6h regimen	Route: intravenous (IV). Dose: 100 mg/kg per dose. Interval: every 6 h. Duration: until delivery.	Rationale: maternal t1/2 approximately 1.2 h; q6h dosing is intended to prevent sub-therapeutic levels.	Jenkins et al. (11); Wiest et al. (7)
Maternal dosing	Option B—FDA 3-bag protocol	Loading dose: 150 mg/kg IV over 1 h. Maintenance 1: 50 mg/kg IV over 4 h. Maintenance 2: 100 mg/kg IV over 16 h.	Protocol used in the Triple I RCT.	Buhimschi et al. (10)
Placental transfer	Cord: maternal NAC ratio	Cord: maternal NAC ratio approximately 1.4.	Finding supports rapid placental transfer.	
Neonatal dosing	Standard postnatal regimen	Route: IV preferred. Dose: 12.5–25 mg/kg per dose, weight-based. Interval: every 12 h. Number of doses: 5 total. Start: approximately 6 h after the last maternal dose.	Oral bioavailability is unknown in neonates.	
Gestational-age adjustment	Preterm neonates <34 weeks	Neonatal t1/2: approximately 7.5 h. Dose: 12.5 mg/kg per dose.	Use q12 h dosing; prolonged neonatal t1/2 requires an extended interval to avoid accumulation.	
Gestational-age adjustment	Near-term neonates >=34 weeks	Neonatal t1/2: approximately 5.1 h. Dose: 25 mg/kg per dose.	Use q12 h dosing; prolonged neonatal t1/2 requires an extended interval to avoid accumulation.	
Safety profile	Human studies	No maternal deaths or severe adverse events; no hemodynamic instability; mild infusion reactions manageable by slowing the infusion rate; no anaphylactoid reactions reported.	No increase in cesarean delivery or PPH; no neonatal hepatic or renal toxicity; normal MRI/MRS at term-equivalent age; no CP or ASD at 4-year follow-up, with small sample size.	
Efficacy outcomes	Triple I RCT	Buhimschi 2021: *n* = 67 dyads. Composite morbidity: 21% vs. 47%; RR 0.45 (95% CI 0.22–0.91), *p* = 0.03. BPD: 3% vs. 32%; RR 0.10 (95% CI 0.01–0.73), *p* = 0.006.	Key reported neonatal morbidity outcomes.	Buhimschi et al. (10)
Efficacy outcomes	Pilot RCT	Jenkins 2016: *n* = 22 mothers and 24 infants. Cerebrovascular coupling was preserved (*p* = 0.0003). IL-1Ra increased, and VEGF decreased (*p*<=0.014).	No RR/CI reported; safety-focused pilot trial.	Jenkins et al. (11)

ASD, autism spectrum disorder; BPD, bronchopulmonary dysplasia; CI, confidence interval; CP, cerebral palsy; GA, gestational age; IL-1Ra, interleukin-1 receptor antagonist; IV, intravenous; MRI/MRS, magnetic resonance imaging/magnetic resonance spectroscopy; NAC, N-acetylcysteine; PPH, postpartum hemorrhage; RCT, randomized controlled trial; RR, risk ratio; VEGF, vascular endothelial growth factor.

Verify all doses, outcomes, and citations against the final manusc*r*ipt references and original study protocols before submission or clinical use.

### Comparison with existing neuroprotective strategies

Current neuroprotective interventions for inflammation-associated preterm injury remain limited. Magnesium sulfate reduces the risk of cerebral palsy in imminent preterm birth, as demonstrated in systematic reviews and meta-analyses, but does not directly target oxidative or inflammatory pathways central to the pathophysiology of chorioamnionitis (5). Alternative neuroprotective approaches, including erythropoietin, calcitriol combination therapies, and melatonin, remain investigational with limited obstetric safety and efficacy data in inflammation-mediated preterm injury (19). Compared with these approaches, NAC offers several practical advantages, including established clinical availability, relatively low cost, known obstetric safety, rapid placental transfer, and multimodal anti-inflammatory and antioxidant activity.

Magnesium sulfate lacks anti-inflammatory potency and does not target the oxidative cascades central to the pathophysiology of chorioamnionitis (20). Melatonin and erythropoietin are neuroprotective but lack obstetric safety data. NAC is a pragmatic addition to routine therapy due to its safety, low cost, and dual antioxidant/anti-inflammatory properties. NAC can be compared to other neuroprotective methods like magnesium sulfate, which reduces the risk of cerebral palsy in imminent preterm birth but lacks direct anti-inflammatory or antioxidant activity and has unproven efficacy in chorioamnionitis (10, 11). Prenatal corticosteroids simply promote lung maturation and are not neuroprotective. Therapeutic hypothermia is contraindicated for premature babies. Erythropoietin has preclinical justification but conflicting findings in human RCTs, and its dose window and timing are unknown. Antioxidant melatonin has inconsistent absorption and limited safety/pregnancy data.

NAC is FDA-approved, has been used in obstetrics for decades to treat acetaminophen toxicity, and targets pathways involved in chorioamnionitis-related damage (glutathione replenishment, cytokine regulation, vascular stabilisation). NAC can be given with magnesium sulfate and corticosteroids to boost neuroprotection rather than replace proven therapy.

### Clinical implementation considerations

- Diagnostic certainty: NAC could be initiated at the time of clinical diagnosis of chorioamnionitis or confirmed Triple I; given its short half-life, rapid initiation is feasible.
- Integration with existing protocols: NAC can be co-administered with magnesium sulfate and antibiotic regimens without known interactions.
- Monitoring: Standard infusion monitoring for rare anaphylactoid reactions is warranted; slow loading dose minimises risk.
- Resource implications: NAC is inexpensive and widely available and could be rapidly adopted if efficacy is confirmed.

### Alignment With current guidelines

No ACOG or SMFM guidance exists on NAC for chorioamnionitis neuroprotection. NAC's safety profile and Triple I RCT signal suggest that local pilot implementation protocols (under research oversight, similar to magnesium sulfate neuroprotection algorithms) with standardised checklists for eligibility, dosing, and monitoring may be appropriate. Table 5 shows NAC dosing reference–chorioamnionitis/triple I.

### Ethical and equity considerations

A chorioamnionitis diagnosis is needed to start NAC, which can be done quickly or slowly depending on resources. If combined with chorioamnionitis diagnostic consistency, NAC at varied facilities could improve neuroprotective care. Informed consent talks must emphasise NAC's exploratory status for this indication, known safety profile, and unclear benefit. NAC is cheap and off-patent, so global access should be possible if it works.

Equity transcends cost. Clinical diagnosis, rapid laboratory turnaround, or amniocentesis for chorioamnionitis relies on hospital-level staffing and location. Low-resource or understaffed settings may delay or neglect clinical chorioamnionitis diagnosis, preventing NAC initiation. Well-equipped tertiary care hospitals can document Triple I and precisely select patients using amniocentesis. Consider point-of-care IL-6 testing, automated sepsis bundles in EHRs, and community hospital training programs to democratize NAC access. Global LMIC procurement requires manufacturing scale-up, cold-chain-free formulations in some regions, and WHO prequalification. Since NAC is off patent, generics may market it cheaply.

NAC is FDA-approved for numerous purposes, but not for pregnancy neuroprotection. In a family facing impending preterm delivery and considerable anguish, professionals should be enthusiastic but balanced and empathic. NAC should be presented as an exploratory but promising medication with few small trials, but no clear proof, and magnesium sulfate and corticosteroids should always be offered during consent negotiations. When chorioamnionitis is suspected but unconfirmed or birth is approaching, and antenatal NAC is limited, shared decision-making is essential.

### Risk–benefit profile and counselling

NAC may reduce severe newborn morbidity, enhance cerebrovascular control, and cut BPD and NEC rates. Known risks are minimal based on existing data; rare anaphylactoid reactions are mitigated with slow infusion and preparedness to give antihistamines. Clinicians should underline NAC's safety in other uses, its exploratory neuroprotection, and the uncertainty of its usefulness when discussing it with families.

### Clinical and research implications

Despite encouraging findings, the current evidence base remains preliminary. Most human studies were single-centre investigations with relatively small sample sizes, limiting statistical power and generalizability. Heterogeneity in diagnostic criteria, NAC dosing regimens, timing of administration, and outcome definitions also restricted direct comparisons across studies. Furthermore, several clinical outcomes were surrogate biomarkers rather than long-term neurodevelopmental endpoints.

Future multicenter randomised controlled trials should therefore employ standardised Triple I diagnostic criteria and correctly identify the variability in chorioamnionitis and Triple I definitions, harmonised NAC dosing protocols, and long-term neurodevelopmental follow-up extending beyond infancy. Important future outcomes should include severe composite neonatal morbidity, cerebral palsy, neurocognitive development, MRI biomarkers, and inflammation-related respiratory outcomes such as BPD. Integration of pharmacokinetic-pharmacodynamic modelling and biomarker-guided dosing strategies may further optimise therapeutic precision.

### Strengths and limitations

This review has several strengths, including integration of human clinical and translational animal evidence, comprehensive database searching, PRISMA-guided methodology, and design-specific risk-of-bias assessment tools. However, several limitations should be acknowledged. The search was limited to English-language publications, and authors were not approached for missing data. The overall number of clinical human studies was small; most studies were single-centre investigations, and substantial methodological heterogeneity precluded quantitative synthesis. The main clinical conclusions are based on one moderate-sized randomised controlled trial (67 dyads), a small mechanistic RCT (22 mothers/24 infants), and associated pharmacokinetic and follow-up studies. Although reported effect sizes, such as the reduction in BPD, are derived from modest samples at a single centre. Additionally, publication bias could not be reliably assessed because of the limited number of included studies. Narrative synthesis was used because NAC dose and outcome measures were clinically heterogeneous among studies, preventing a quantitative meta-analysis.

## Conclusion

N-acetylcysteine demonstrates biologically plausible neuroprotective potential in pregnancies complicated by maternal chorioamnionitis and Triple I. Current evidence from both animal and human studies suggests possible benefits in reducing severe neonatal morbidity, preserving cerebrovascular regulation, attenuating inflammatory injury, and lowering bronchopulmonary dysplasia risk without major safety concerns. However, some studies showed no or negative effects. Existing evidence remains insufficient for routine clinical implementation because of small sample sizes, heterogeneous protocols, and limited long-term outcome data. NAC should be framed as a promising candidate, but one that requires validation through Large, adequately powered, coordinated multicenter randomised controlled trials with long-term neurodevelopmental outcomes before NAC can be recommended as standard adjunctive neuroprotective therapy in maternal chorioamnionitis.

## Data Availability

The raw data supporting the conclusions of this article will be made available by the authors without undue reservation.

## Glossary

ACOG: american college of obstetricians and gynecologists
ASD: autistic spectrum disorders
BPD: bronchopulmonary dysplasia
CI: confidence interval
CNS: central nervous system
CP: cerebral palsy
DTI: diffusion tensor imaging
EHRs: electronic health records
FDA: food and drug administration
FIRS: fetal inflammatory response syndrome
GA: gestational age
GRADE: grading of recommendations assessment, development and evaluation
GSH: reduced glutathione
GSSG: oxidised glutathione
HDAC2: histone deacetylase 2
HIE: hypoxic-ischemic encephalopathy
IL: Interleukin
IV: Intravenous
IVH: intraventricular haemorrhage
LPS: lipopolysaccharide
mCA: maternal chorioamnionitis
MBP: myelin basic protein
MRI: magnetic resonance imaging
MRS: magnetic resonance spectroscopy
NAC: N-Acetylcysteine
NADPH: nicotinamide adenine dinucleotide phosphate
NEC: necrotising enterocolitis
NF-*κ*B: nuclear factor kappa B
NF-H: neurofilament heavy chain
NICU: neonatal intensive care unit
NIRS: near-infrared spectroscopy
nNOS: neuronal nitric oxide synthase
NRCT: non-randomised controlled trial
PK: pharmacokinetics
PLP: proteolipid protein
PRISMA: preferred reporting items for systematic reviews and meta-analyses
RCT: randomized controlled trial
RoB: risk of bias
ROP: retinopathy of prematurity
ROS: reactive oxygen species
PVL: periventricular leukomalacia
SMFM: society for maternal-fetal medicine
(t½): Neonatal half-life
TNF-α: tumour necrosis factor alpha
Triple I: Intrauterine Infection or Inflammation
Vd: Volume of distribution
VEGF: vascular endothelial growth factor
WMI: white matter injury
WHO: world health organization
RoB 2: risk of bias 2
ROBINS-I: risk of bias in non-randomised studies of interventions
SYRCLE: systematic review centre for laboratory animal experimentation
